# Health Tract, No. 276

**Published:** 1867-02

**Authors:** 


### TWO PICTURES.

"I have finished the work which thou gavest me to do," said the Savior of Mankind, just before he was "carried up into heaven." The legitimate inference is that every man has his allotted task to perform, and that as soon as it is done he passes on to the Judgement. Some have more assigned to them than others, according to their station, their capacities and their opportunities. How much some do, how little others, is amazing. But no one can doubt that the actual amount of good-doing of any individual would in any given case, be increased, or done better, or in a shorter time, if the body was in vigorous health. Hence every christian man whose heart is full of a desire to do as much as he can for HIM who has done so much for him, should study and practice the means of keeping himself well.

One man is born to poverty and obscurity, yet rises to benefit and bless multitudes; another born to large wealth, or good family and an honorable name, a child of "great expectations," lives and dies, but 'leaves no sign,' that he ever existed for any good. The reason is, the poor boy is obliged to exert himself early for a living; this exercise calls out his energies; these grow by use; he sees it, feels it, and forthwith becomes conscious of a power within him which it is a pleasure to wield; then comes self-reliance and an ambition for greater things; and he wins. But in the prosecution of these things both mind and body have been exercised steadily, and the result is vigorous, manly health, which by this time has become a power for 'greater things than these,' and a power, too, which "lives to a good old age."

The petted child of family and fortune, having bread supplied to his hand, and soon learning that enough has been provided to last him a lifetime, very naturally yields to a lazy, self-indulgent life; his study is not how he may get, but how he may enjoy. At first he runs the rounds of superficial pleasures, sipping from each flower of passion as he passes along; now yielding to one self-indulgence, then another; next come the more positive animal appetites, then the cigar, the glass; gaming and gluttony follow in the train, and before he knows it he is regarded as a gourmand, a roue and a sot; money spent, position sacrificed, character stained, health ruined, life blasted, he lays himself down to die, forsaken of friends, left to remorseful memories of opportunities lost and talents desecrated; meanwhile disease preys on his vitals, darting its fierce and fiery pains through every limb, stretching, tearing, crushing every nerve; no moment of repose; no drop of water; no smile from other face; no touch from other fingers; no kindly word from other lips; he writhes in wretchedness, a ruined wretch — a wasted life, a lost soul. Two pictures, young man; it is in your power to choose which you will sit to! What is your election?

